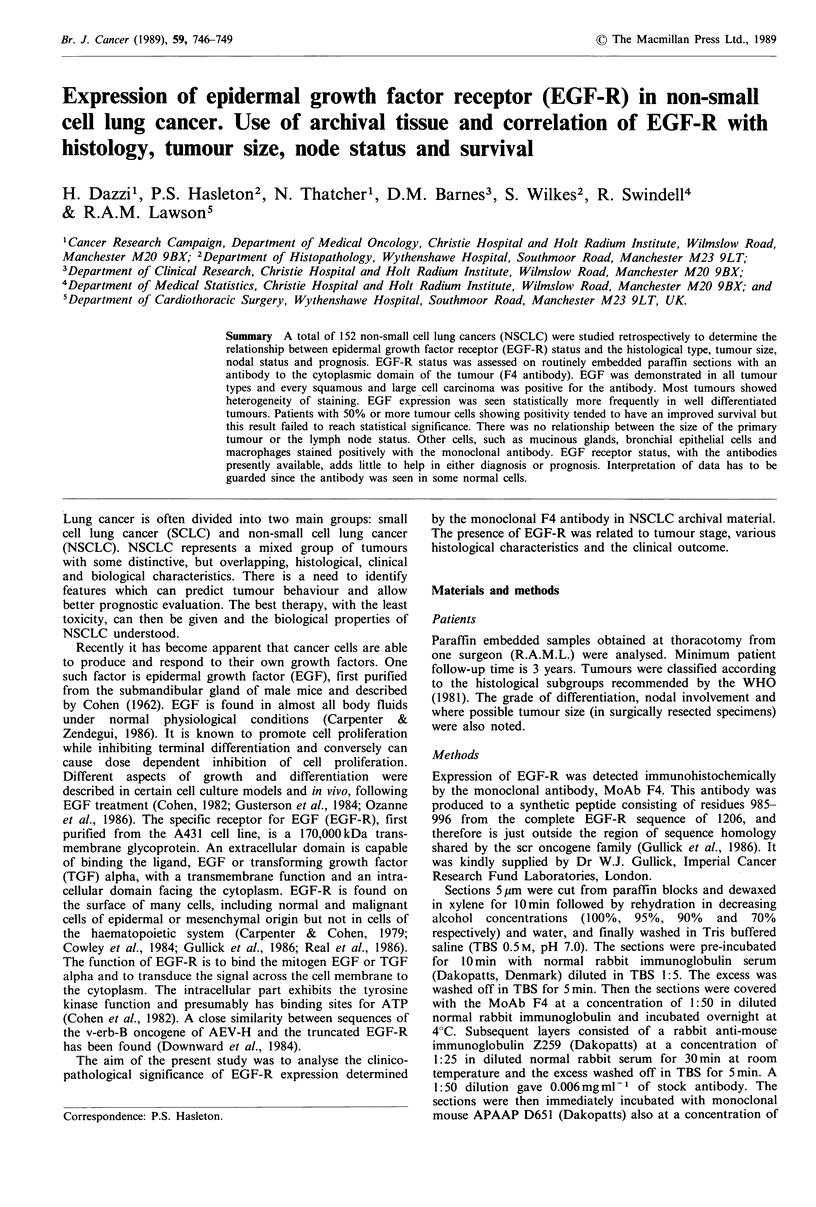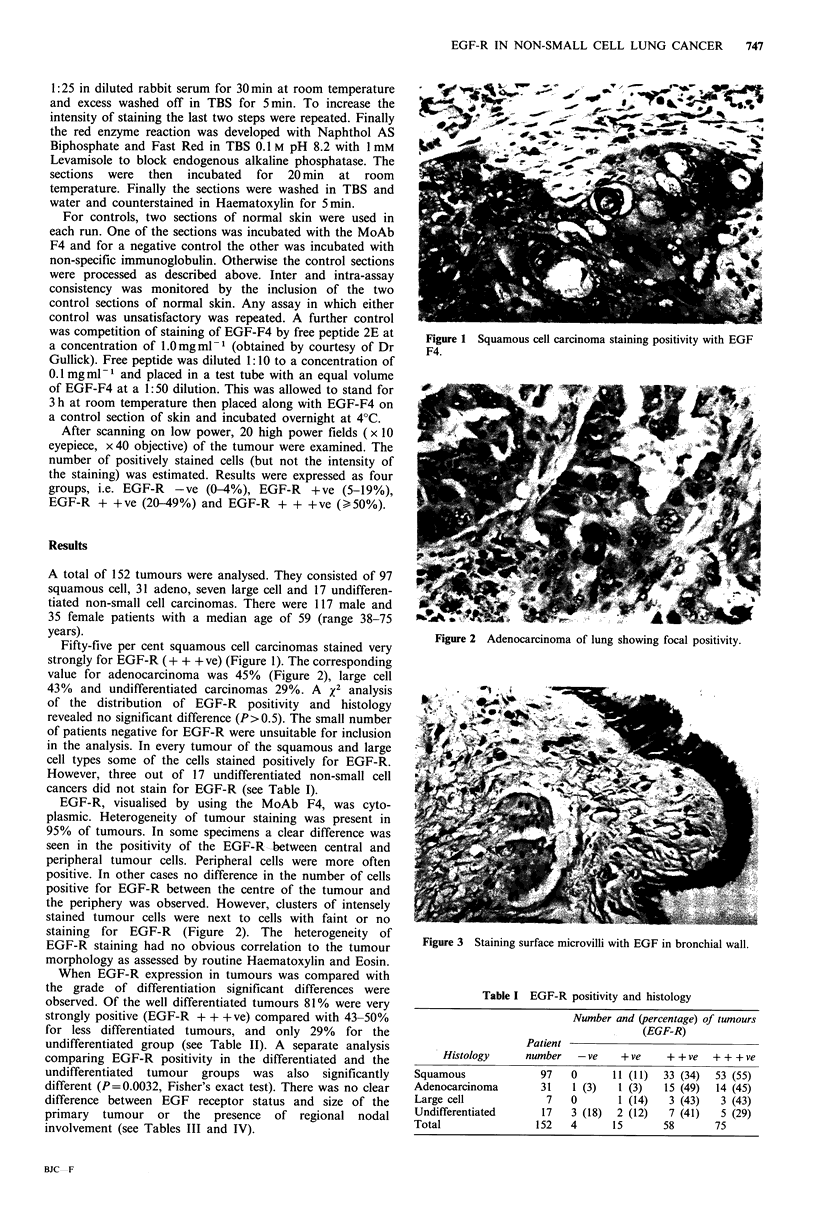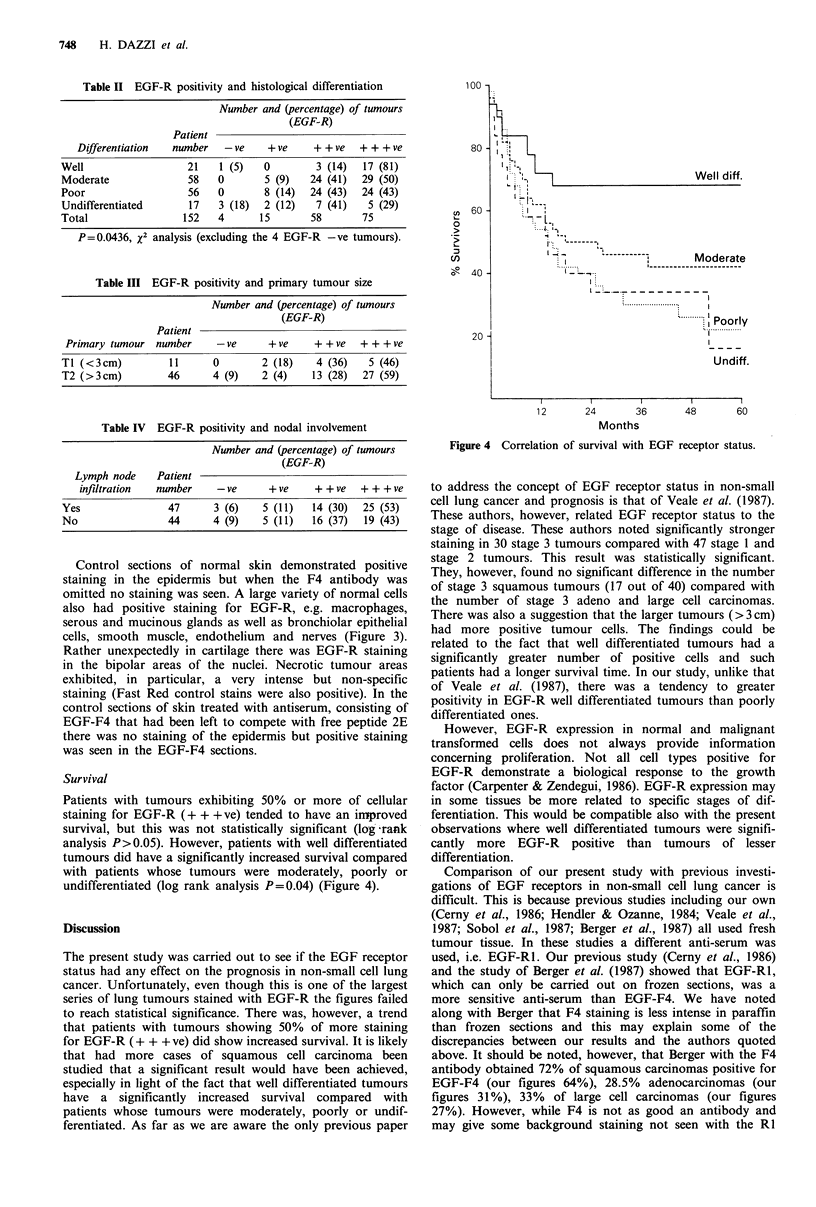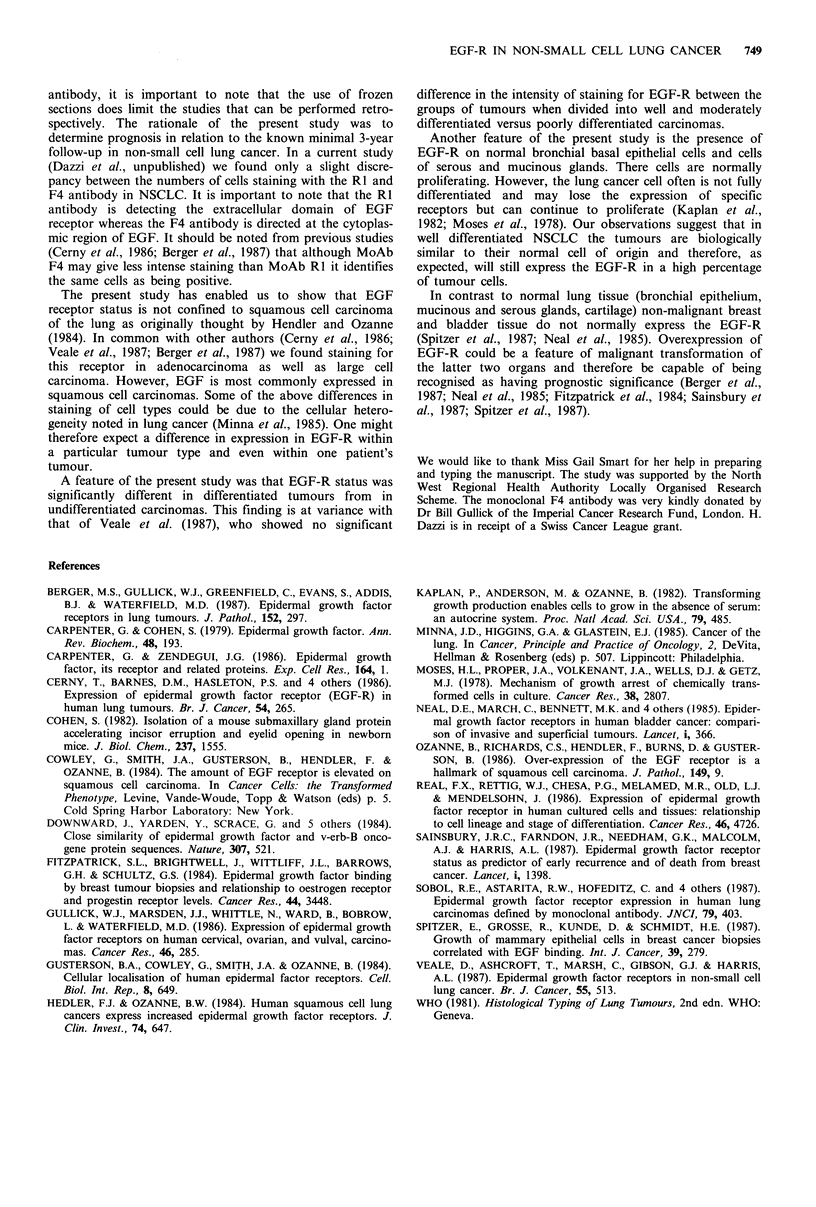# Expression of epidermal growth factor receptor (EGF-R) in non-small cell lung cancer. Use of archival tissue and correlation of EGF-R with histology, tumour size, node status and survival.

**DOI:** 10.1038/bjc.1989.156

**Published:** 1989-05

**Authors:** H. Dazzi, P. S. Hasleton, N. Thatcher, D. M. Barnes, S. Wilkes, R. Swindell, R. A. Lawson

**Affiliations:** Department of Medical Oncology, Christie Hospital, Manchester, UK.

## Abstract

**Images:**


*Br. J. Cancer* (1989), **59**, 746–749 © The Macmillan Press Ltd., 1989

### Expression of epidermal growth factor receptor (EGF-R) in non-small cell lung cancer. Use of archival tissue and correlation of EGF-R with histology, tumour size, node status and survival

H. Dazzi[1], P.S. Hasleton[2], N. Thatcher[1], D.M. Barnes[3], S. Wilkes[2], R. Swindell[4] & R.A.M. Lawson[5]

[1]*Cancer Research Campaign, Department of Medical Oncology, Christie Hospital and Holt Radium Institute, Wilmslow Road, Manchester M20 9BX;* [2]*Department of Histopathology, Wythenshawe Hospital, Southmoor Road, Manchester M23 9LT;* [3]*Department of Clinical Research, Christie Hospital and Holt Radium Institute, Wilmslow Road, Manchester M20 9BX;* [4]*Department of Medical Statistics, Christie Hospital and Holt Radium Institute, Wilmslow Road, Manchester M20 9BX; and* [5]*Department of Cardiothoracic Surgery, Wythenshawe Hospital, Southmoor Road, Manchester M23 9LT, UK.*

**Summary** A total of 152 non-small cell lung cancers (NSCLC) were studied retrospectively to determine the relationship between epidermal growth factor receptor (EGF-R) status and the histological type, tumour size, nodal status and prognosis. EGF-R status was assessed on routinely embedded paraffin sections with an antibody to the cytoplasmic domain of the tumour (F4 antibody). EGF was demonstrated in all tumour types and every squamous and large cell carcinoma was positive for the antibody. Most tumours showed heterogeneity of staining. EGF expression was seen statistically more frequently in well differentiated tumours. Patients with 50% or more tumour cells showing positivity tended to have an improved survival but this result failed to reach statistical significance. There was no relationship between the size of the primary tumour or the lymph node status. Other cells, such as mucinous glands, bronchial epithelial cells and macrophages stained positively with the monoclonal antibody. EGF receptor status, with the antibodies presently available, adds little to help in either diagnosis or prognosis. Interpretation of data has to be guarded since the antibody was seen in some normal cells.

Lung cancer is often divided into two main groups: small cell lung cancer (SCLC) and non-small cell lung cancer (NSCLC). NSCLC represents a mixed group of tumours with some distinctive, but overlapping, histological, clinical and biological characteristics. There is a need to identify features which can predict tumour behaviour and allow better prognostic evaluation. The best therapy, with the least toxicity, can then be given and the biological properties of NSCLC understood.

Recently it has become apparent that cancer cells are able to produce and respond to their own growth factors. One such factor is epidermal growth factor (EGF), first purified from the submandibular gland of male mice and described by Cohen (1962). EGF is found in almost all body fluids under normal physiological conditions (Carpenter & Zendegui, 1986). It is known to promote cell proliferation while inhibiting terminal differentiation and conversely can cause dose dependent inhibition of cell proliferation. Different aspects of growth and differentiation were described in certain cell culture models and *in vivo*, following EGF treatment (Cohen, 1982; Gusterson *et al.*, 1984; Ozanne *et al.*, 1986). The specific receptor for EGF (EGF-R), first purified from the A431 cell line, is a 170,000 kDa transmembrane glycoprotein. An extracellular domain is capable of binding the ligand, EGF or transforming growth factor (TGF) alpha, with a transmembrane function and an intracellular domain facing the cytoplasm. EGF-R is found on the surface of many cells, including normal and malignant cells of epidermal or mesenchymal origin but not in cells of the haematopoietic system (Carpenter & Cohen, 1979; Cowley *et al.*, 1984; Gullick *et al.*, 1986; Real *et al.*, 1986). The function of EGF-R is to bind the mitogen EGF or TGF alpha and to transduce the signal across the cell membrane to the cytoplasm. The intracellular part exhibits the tyrosine kinase function and presumably has binding sites for ATP (Cohen *et al.*, 1982). A close similarity between sequences of the v-erb-B oncogene of AEV-H and the truncated EGF-R has been found (Downward *et al.*, 1984).

The aim of the present study was to analyse the clinicopathological significance of EGF-R expression determined by the monoclonal F4 antibody in NSCLC archival material. The presence of EGF-R was related to tumour stage, various histological characteristics and the clinical outcome.

Correspondence: P.S. Hasleton.

### Materials and methods

### *Patients*

Paraffin embedded samples obtained at thoracotomy from one surgeon (R.A.M.L.) were analysed. Minimum patient follow-up time is 3 years. Tumours were classified according to the histological subgroups recommended by the WHO (1981). The grade of differentiation, nodal involvement and where possible tumour size (in surgically resected specimens) were also noted.

### *Methods*

Expression of EGF-R was detected immunohistochemically by the monoclonal antibody, MoAb F4. This antibody was produced to a synthetic peptide consisting of residues 985–996 from the complete EGF-R sequence of 1206, and therefore is just outside the region of sequence homology shared by the scr oncogene family (Gullick *et al.*, 1986). It was kindly supplied by Dr W.J. Gullick, Imperial Cancer Research Fund Laboratories, London.

Sections 5 $\mu$m were cut from paraffin blocks and dewaxed in xylene for 10 min followed by rehydration in decreasing alcohol concentrations (100%, 95%, 90% and 70% respectively) and water, and finally washed in Tris buffered saline (TBS 0.5 M, pH 7.0). The sections were pre-incubated for 10 min with normal rabbit immunoglobulin serum (Dakopatts, Denmark) diluted in TBS 1:5. The excess was washed off in TBS for 5 min. Then the sections were covered with the MoAb F4 at a concentration of 1:50 in diluted normal rabbit immunoglobulin and incubated overnight at 4°C. Subsequent layers consisted of a rabbit anti-mouse immunoglobulin Z259 (Dakopatts) at a concentration of 1:25 in diluted normal rabbit serum for 30 min at room temperature and the excess washed off in TBS for 5 min. A 1:50 dilution gave 0.006 mg ml$^{-1}$ of stock antibody. The sections were then immediately incubated with monoclonal mouse APAAP D651 (Dakopatts) also at a concentration of

1:25 in diluted rabbit serum for 30 min at room temperature and excess washed off in TBS for 5 min. To increase the intensity of staining the last two steps were repeated. Finally the red enzyme reaction was developed with Naphthol AS Biphosphate and Fast Red in TBS 0.1 M pH 8.2 with 1 mM Levamisole to block endogenous alkaline phosphatase. The sections were then incubated for 20 min at room temperature. Finally the sections were washed in TBS and water and counterstained in Haematoxylin for 5 min.

For controls, two sections of normal skin were used in each run. One of the sections was incubated with the MoAb F4 and for a negative control the other was incubated with non-specific immunoglobulin. Otherwise the control sections were processed as described above. Inter and intra-assay consistency was monitored by the inclusion of the two control sections of normal skin. Any assay in which either control was unsatisfactory was repeated. A further control was competition of staining of EGF-F4 by free peptide 2E at a concentration of 1.0 mg ml$^{-1}$ (obtained by courtesy of Dr Gullick). Free peptide was diluted 1:10 to a concentration of 0.1 mg ml$^{-1}$ and placed in a test tube with an equal volume of EGF-F4 at a 1:50 dilution. This was allowed to stand for 3 h at room temperature then placed along with EGF-F4 on a control section of skin and incubated overnight at 4°C.

After scanning on low power, 20 high power fields (×10 eyepiece, ×40 objective) of the tumour were examined. The number of positively stained cells (but not the intensity of the staining) was estimated. Results were expressed as four groups, i.e. EGF-R −ve (0–4%), EGF-R +ve (5–19%), EGF-R + +ve (20–49%) and EGF-R + + +ve (≥50%).

### Results

A total of 152 tumours were analysed. They consisted of 97 squamous cell, 31 adeno, seven large cell and 17 undifferentiated non-small cell carcinomas. There were 117 male and 35 female patients with a median age of 59 (range 38–75 years).

Fifty-five per cent squamous cell carcinomas stained very strongly for EGF-R (+ + +ve) (Figure 1). The corresponding value for adenocarcinoma was 45% (Figure 2), large cell 43% and undifferentiated carcinomas 29%. A $\chi^2$ analysis of the distribution of EGF-R positivity and histology revealed no significant difference ($P>0.5$). The small number of patients negative for EGF-R were unsuitable for inclusion in the analysis. In every tumour of the squamous and large cell types some of the cells stained positively for EGF-R. However, three out of 17 undifferentiated non-small cell cancers did not stain for EGF-R (see Table I).

EGF-R, visualised by using the MoAb F4, was cytoplasmic. Heterogeneity of tumour staining was present in 95% of tumours. In some specimens a clear difference was seen in the positivity of the EGF-R between central and peripheral tumour cells. Peripheral cells were more often positive. In other cases no difference in the number of cells positive for EGF-R between the centre of the tumour and the periphery was observed. However, clusters of intensely stained tumour cells were next to cells with faint or no staining for EGF-R (Figure 2). The heterogeneity of EGF-R staining had no obvious correlation to the tumour morphology as assessed by routine Haematoxylin and Eosin.

When EGF-R expression in tumours was compared with the grade of differentiation significant differences were observed. Of the well differentiated tumours 81% were very strongly positive (EGF-R + + +ve) compared with 43–50% for less differentiated tumours, and only 29% for the undifferentiated group (see Table II). A separate analysis comparing EGF-R positivity in the differentiated and the undifferentiated tumour groups was also significantly different ($P=0.0032$, Fisher's exact test). There was no clear difference between EGF receptor status and size of the primary tumour or the presence of regional nodal involvement (see Tables III and IV).

**Figure 1** Squamous cell carcinoma staining positivity with EGF F4.

**Figure 2** Adenocarcinoma of lung showing focal positivity.

**Figure 3** Staining surface microvilli with EGF in bronchial wall.

**Table I** EGF-R positivity and histology

| *Histology* | *Patient number* | *Number and (percentage) of tumours (EGF-R)* | | | |
|---|---|---|---|---|---|
| | | *−ve* | *+ve* | *+ +ve* | *+ + +ve* |
| Squamous | 97 | 0 | 11 (11) | 33 (34) | 53 (55) |
| Adenocarcinoma | 31 | 1 (3) | 1 (3) | 15 (49) | 14 (45) |
| Large cell | 7 | 0 | 1 (14) | 3 (43) | 3 (43) |
| Undifferentiated | 17 | 3 (18) | 2 (12) | 7 (41) | 5 (29) |
| Total | 152 | 4 | 15 | 58 | 75 |

**Table II** EGF-R positivity and histological differentiation

| Differentiation | Patient number | Number and (percentage) of tumours (EGF-R) | | | |
|---|---|---|---|---|---|
| | | −ve | +ve | + +ve | + + +ve |
| Well | 21 | 1 (5) | 0 | 3 (14) | 17 (81) |
| Moderate | 58 | 0 | 5 (9) | 24 (41) | 29 (50) |
| Poor | 56 | 0 | 8 (14) | 24 (43) | 24 (43) |
| Undifferentiated | 17 | 3 (18) | 2 (12) | 7 (41) | 5 (29) |
| Total | 152 | 4 | 15 | 58 | 75 |

$P = 0.0436$, $\chi^2$ analysis (excluding the 4 EGF-R −ve tumours).

**Table III** EGF-R positivity and primary tumour size

| Primary tumour | Patient number | Number and (percentage) of tumours (EGF-R) | | | |
|---|---|---|---|---|---|
| | | −ve | +ve | + +ve | + + +ve |
| T1 (<3 cm) | 11 | 0 | 2 (18) | 4 (36) | 5 (46) |
| T2 (>3 cm) | 46 | 4 (9) | 2 (4) | 13 (28) | 27 (59) |

**Table IV** EGF-R positivity and nodal involvement

| Lymph node infiltration | Patient number | Number and (percentage) of tumours (EGF-R) | | | |
|---|---|---|---|---|---|
| | | −ve | +ve | + +ve | + + +ve |
| Yes | 47 | 3 (6) | 5 (11) | 14 (30) | 25 (53) |
| No | 44 | 4 (9) | 5 (11) | 16 (37) | 19 (43) |

Control sections of normal skin demonstrated positive staining in the epidermis but when the F4 antibody was omitted no staining was seen. A large variety of normal cells also had positive staining for EGF-R, e.g. macrophages, serous and mucinous glands as well as bronchiolar epithelial cells, smooth muscle, endothelium and nerves (Figure 3). Rather unexpectedly in cartilage there was EGF-R staining in the bipolar areas of the nuclei. Necrotic tumour areas exhibited, in particular, a very intense but non-specific staining (Fast Red control stains were also positive). In the control sections of skin treated with antiserum, consisting of EGF-F4 that had been left to compete with free peptide 2E there was no staining of the epidermis but positive staining was seen in the EGF-F4 sections.

*Survival*

Patients with tumours exhibiting 50% or more of cellular staining for EGF-R (+ + +ve) tended to have an improved survival, but this was not statistically significant (log rank analysis $P > 0.05$). However, patients with well differentiated tumours did have a significantly increased survival compared with patients whose tumours were moderately, poorly or undifferentiated (log rank analysis $P = 0.04$) (Figure 4).

### Discussion

The present study was carried out to see if the EGF receptor status had any effect on the prognosis in non-small cell lung cancer. Unfortunately, even though this is one of the largest series of lung tumours stained with EGF-R the figures failed to reach statistical significance. There was, however, a trend that patients with tumours showing 50% of more staining for EGF-R (+ + +ve) did show increased survival. It is likely that had more cases of squamous cell carcinoma been studied that a significant result would have been achieved, especially in light of the fact that well differentiated tumours have a significantly increased survival compared with patients whose tumours were moderately, poorly or undifferentiated. As far as we are aware the only previous paper

**Figure 4** Correlation of survival with EGF receptor status.

to address the concept of EGF receptor status in non-small cell lung cancer and prognosis is that of Veale *et al.* (1987). These authors, however, related EGF receptor status to the stage of disease. These authors noted significantly stronger staining in 30 stage 3 tumours compared with 47 stage 1 and stage 2 tumours. This result was statistically significant. They, however, found no significant difference in the number of stage 3 squamous tumours (17 out of 40) compared with the number of stage 3 adeno and large cell carcinomas. There was also a suggestion that the larger tumours (>3 cm) had more positive tumour cells. The findings could be related to the fact that well differentiated tumours had a significantly greater number of positive cells and such patients had a longer survival time. In our study, unlike that of Veale *et al.* (1987), there was a tendency to greater positivity in EGF-R well differentiated tumours than poorly differentiated ones.

However, EGF-R expression in normal and malignant transformed cells does not always provide information concerning proliferation. Not all cell types positive for EGF-R demonstrate a biological response to the growth factor (Carpenter & Zendegui, 1986). EGF-R expression may in some tissues be more related to specific stages of differentiation. This would be compatible also with the present observations where well differentiated tumours were significantly more EGF-R positive than tumours of lesser differentiation.

Comparison of our present study with previous investigations of EGF receptors in non-small cell lung cancer is difficult. This is because previous studies including our own (Cerny *et al.*, 1986; Hendler & Ozanne, 1984; Veale *et al.*, 1987; Sobol *et al.*, 1987; Berger *et al.*, 1987) all used fresh tumour tissue. In these studies a different anti-serum was used, i.e. EGF-R1. Our previous study (Cerny *et al.*, 1986) and the study of Berger *et al.* (1987) showed that EGF-R1, which can only be carried out on frozen sections, was a more sensitive anti-serum than EGF-F4. We have noted along with Berger that F4 staining is less intense in paraffin than frozen sections and this may explain some of the discrepancies between our results and the authors quoted above. It should be noted, however, that Berger with the F4 antibody obtained 72% of squamous carcinomas positive for EGF-F4 (our figures 64%), 28.5% adenocarcinomas (our figures 31%), 33% of large cell carcinomas (our figures 27%). However, while F4 is not as good an antibody and may give some background staining not seen with the R1

antibody, it is important to note that the use of frozen sections does limit the studies that can be performed retrospectively. The rationale of the present study was to determine prognosis in relation to the known minimal 3-year follow-up in non-small cell lung cancer. In a current study (Dazzi *et al.*, unpublished) we found only a slight discrepancy between the numbers of cells staining with the R1 and F4 antibody in NSCLC. It is important to note that the R1 antibody is detecting the extracellular domain of EGF receptor whereas the F4 antibody is directed at the cytoplasmic region of EGF. It should be noted from previous studies (Cerny *et al.*, 1986; Berger *et al.*, 1987) that although MoAb F4 may give less intense staining than MoAb R1 it identifies the same cells as being positive.

The present study has enabled us to show that EGF receptor status is not confined to squamous cell carcinoma of the lung as originally thought by Hendler and Ozanne (1984). In common with other authors (Cerny *et al.*, 1986; Veale *et al.*, 1987; Berger *et al.*, 1987) we found staining for this receptor in adenocarcinoma as well as large cell carcinoma. However, EGF is most commonly expressed in squamous cell carcinomas. Some of the above differences in staining of cell types could be due to the cellular heterogeneity noted in lung cancer (Minna *et al.*, 1985). One might therefore expect a difference in expression in EGF-R within a particular tumour type and even within one patient's tumour.

A feature of the present study was that EGF-R status was significantly different in differentiated tumours from in undifferentiated carcinomas. This finding is at variance with that of Veale *et al.* (1987), who showed no significant difference in the intensity of staining for EGF-R between the groups of tumours when divided into well and moderately differentiated versus poorly differentiated carcinomas.

Another feature of the present study is the presence of EGF-R on normal bronchial basal epithelial cells and cells of serous and mucinous glands. There cells are normally proliferating. However, the lung cancer cell often is not fully differentiated and may lose the expression of specific receptors but can continue to proliferate (Kaplan *et al.*, 1982; Moses *et al.*, 1978). Our observations suggest that in well differentiated NSCLC the tumours are biologically similar to their normal cell of origin and therefore, as expected, will still express the EGF-R in a high percentage of tumour cells.

In contrast to normal lung tissue (bronchial epithelium, mucinous and serous glands, cartilage) non-malignant breast and bladder tissue do not normally express the EGF-R (Spitzer *et al.*, 1987; Neal *et al.*, 1985). Overexpression of EGF-R could be a feature of malignant transformation of the latter two organs and therefore be capable of being recognised as having prognostic significance (Berger *et al.*, 1987; Neal *et al.*, 1985; Fitzpatrick *et al.*, 1984; Sainsbury *et al.*, 1987; Spitzer *et al.*, 1987).

We would like to thank Miss Gail Smart for her help in preparing and typing the manuscript. The study was supported by the North West Regional Health Authority Locally Organised Research Scheme. The monoclonal F4 antibody was very kindly donated by Dr Bill Gullick of the Imperial Cancer Research Fund, London. H. Dazzi is in receipt of a Swiss Cancer League grant.

### References

BERGER, M.S., GULLICK, W.J., GREENFIELD, C., EVANS, S., ADDIS, B.J. & WATERFIELD, M.D. (1987). Epidermal growth factor receptors in lung tumours. *J. Pathol.*, **152**, 297.

CARPENTER, G. & COHEN, S. (1979). Epidermal growth factor. *Ann. Rev. Biochem.*, **48**, 193.

CARPENTER, G. & ZENDEGUI, J.G. (1986). Epidermal growth factor, its receptor and related proteins. *Exp. Cell Res.*, **164**, 1.

CERNY, T., BARNES, D.M., HASLETON, P.S. and 4 others (1986). Expression of epidermal growth factor receptor (EGF-R) in human lung tumours. *Br. J. Cancer*, **54**, 265.

COHEN, S. (1982). Isolation of a mouse submaxillary gland protein accelerating incisor erruption and eyelid opening in newborn mice. *J. Biol. Chem.*, **237**, 1555.

COWLEY, G., SMITH, J.A., GUSTERSON, B., HENDLER, F. & OZANNE, B. (1984). The amount of EGF receptor is elevated on squamous cell carcinoma. In *Cancer Cells: the Transformed Phenotype*, Levine, Vande-Woude, Topp & Watson (eds) p. 5. Cold Spring Harbor Laboratory: New York.

DOWNWARD, J., YARDEN, Y., SCRACE, G. and 5 others (1984). Close similarity of epidermal growth factor and v-erb-B oncogene protein sequences. *Nature*, **307**, 521.

FITZPATRICK, S.L., BRIGHTWELL, J., WITTLIFF, J.L., BARROWS, G.H. & SCHULTZ, G.S. (1984). Epidermal growth factor binding by breast tumour biopsies and relationship to oestrogen receptor and progestin receptor levels. *Cancer Res.*, **44**, 3448.

GULLICK, W.J., MARSDEN, J.J., WHITTLE, N., WARD, B., BOBROW, L. & WATERFIELD, M.D. (1986). Expression of epidermal growth factor receptors on human cervical, ovarian, and vulval, carcinomas. *Cancer Res.*, **46**, 285.

GUSTERSON, B.A., COWLEY, G., SMITH, J.A. & OZANNE, B. (1984). Cellular localisation of human epidermal factor receptors. *Cell. Biol. Int. Rep.*, **8**, 649.

HEDLER, F.J. & OZANNE, B.W. (1984). Human squamous cell lung cancers express increased epidermal growth factor receptors. *J. Clin. Invest.*, **74**, 647.

KAPLAN, P., ANDERSON, M. & OZANNE, B. (1982). Transforming growth production enables cells to grow in the absence of serum: an autocrine system. *Proc. Natl Acad. Sci. USA.*, **79**, 485.

MINNA, J.D., HIGGINS, G.A. & GLASTEIN, E.J. (1985). Cancer of the lung. In *Cancer, Principle and Practice of Oncology, 2*, DeVita, Hellman & Rosenberg (eds) p. 507. Lippincott: Philadelphia.

MOSES, H.L., PROPER, J.A., VOLKENANT, J.A., WELLS, D.J. & GETZ, M.J. (1978). Mechanism of growth arrest of chemically transformed cells in culture. *Cancer Res.*, **38**, 2807.

NEAL, D.E., MARCH, C., BENNETT, M.K. and 4 others (1985). Epidermal growth factor receptors in human bladder cancer: comparison of invasive and superficial tumours. *Lancet*, **i**, 366.

OZANNE, B., RICHARDS, C.S., HENDLER, F., BURNS, D. & GUSTERSON, B. (1986). Over-expression of the EGF receptor is a hallmark of squamous cell carcinoma. *J. Pathol.*, **149**, 9.

REAL, F.X., RETTIG, W.J., CHESA, P.G., MELAMED, M.R., OLD, L.J. & MENDELSOHN, J. (1986). Expression of epidermal growth factor receptor in human cultured cells and tissues: relationship to cell lineage and stage of differentiation. *Cancer Res.*, **46**, 4726.

SAINSBURY, J.R.C., FARNDON, J.R., NEEDHAM, G.K., MALCOLM, A.J. & HARRIS, A.L. (1987). Epidermal growth factor receptor status as predictor of early recurrence and of death from breast cancer. *Lancet*, **i**, 1398.

SOBOL, R.E., ASTARITA, R.W., HOFEDITZ, C. and 4 others (1987). Epidermal growth factor receptor expression in human lung carcinomas defined by monoclonal antibody. *JNCI*, **79**, 403.

SPITZER, E., GROSSE, R., KUNDE, D. & SCHMIDT, H.E. (1987). Growth of mammary epithelial cells in breast cancer biopsies correlated with EGF binding. *Int. J. Cancer*, **39**, 279.

VEALE, D., ASHCROFT, T., MARSH, C., GIBSON, G.J. & HARRIS, A.L. (1987). Epidermal growth factor receptors in non-small cell lung cancer. *Br. J. Cancer*, **55**, 513.

WHO (1981). *Histological Typing of Lung Tumours*, 2nd edn. WHO: Geneva.